# Fecal microbiota transplantation for severe clostridium difficile infection after left ventricular assist device implantation: a case control study and concise review on the local and regional therapies

**DOI:** 10.1186/s12879-016-1571-6

**Published:** 2016-05-27

**Authors:** Zeina Z. Berro, Righab H. Hamdan, Israa H. Dandache, Mohamad N. Saab, Hussein H. Karnib, Mahmoud H. Younes

**Affiliations:** Gastroentorology Department, Al Rassoul Al Aazam Hospital, Beirut, Lebanon; Cardiology Department, Beirut Cardiac Institute, Beirut, Lebanon; Cardiothoracic Surgery Department, Beirut Cardiac Institute, Beirut, Lebanon; Medical Research Center, Al Rassoul Al Aazam Hospital, Beirut, Lebanon

**Keywords:** Severe Clostridium difficile_1_, Left ventricular assist device implantation_2_, Fecal microbiota transplantation_3_, Pseudomembranous colitis_4_, Therapy_5_

## Abstract

**Background:**

We report herein a case of fecal microbiota transplantation (FMT) used for severe *Clostridium difficile* infection for a 65-year-old Lebanese man who underwent left ventricular assist device implantation. To the best of our knowledge this is the first case report from Lebanon and the region presenting such technique.

**Case presentation:**

The patient experienced diarrhea and rectal bleeding and was diagnosed of pseudomembranous colitis (PMC). His condition failed to improve on maximal pharmacological therapy. Protocolectomy, an invasive operation consisting in resection of the entire colon and rectum seemed to be the last resort before the patient responded to FMT given through gastroscopy.

**Conclusion:**

Despite the increasing experience with FMT for *C. difficile* infection, published evidence in severe related cases from this region is very limited. Hence, we promote adjunctive FMT, an effective noninvasive method, to be considered as a promising early treatment option in severe *C. difficile* infection.

## Background

*Clostridium difficile*, a ubiquitous spore-forming Gram positive anaerobic bacillus, is the leading cause of antibiotic associated colitis which is among the most common infections implicated in increased-stay, morbidity and mortality of hospitalized patients [1, 2]. Inflammation, apoptosis and necrosis of intestinal cells are attributed to the disruption of the intestinal microbial equilibrium, which opens the floor for C. *difficile* to colonize and produce potent enterotoxins and cytotoxins [3]. Over the past few years, there has been an increasing emergence of the hypervirulent and hyperepidemic strain NAP1/B1/027 resulting in severe outbreaks and causing nosocomial infectious diarrhea [4–7]. The European C. *difficile* infection study (ECDIS) findings show that one in 10 cases of C. *difficile* infection is either transferred to intensive care unit, or necessitates colectomy, or dies [2]. Several antimicrobial treatment including metronidazole and vancomycin are approved for clinical use and are still recommended by many studies as the treatment of choice for serious infections [1]. Recently, with the recurrence and failure of classical treatments, new therapeutic strategies became available such as the novel US Food and Drug Administration (FDA) approved antimicrobial agent fidaxomicin, immunoglobulins and toxin chelators (e.g., cholestyramine, colestipol, tolevamer) along with a reevaluation of the conventional treatments with new recommendations for their use [5]. Another reported optional treatment for severe C. *difficile* is FMT, which constitutes restoration of the microbial flora in the lower gastrointestinal tract through the instilment of feces from healthy donors [8–10]. Although there is supporting evidence from different studies proving successful symptomatic resolution within 24 h of the procedure, the infectious diseases society of America (IDSA) and the European society of clinical microbiology and infectious diseases (ESCMID) guidelines concerning FMT recommends that it should be considered only when there is recurrence and failure of antibiotic therapy [3, 11, 12]. Few studies indicate a potential therapeutic role for FMT in extra-intestinal disorders correlated with gut microbiota, such as cardiovascular disease, multiple sclerosis, colorectal cancer and others [13, 14]. Our manuscript describes a FMT case, performed against severe *C. difficile* infection for an open heart surgery patient who underwent left ventricular assist device implantation (LVAD). This is the first case report from Lebanon and the region presenting such technique. Altogether, FMT holds promise for reducing antibiotic use and expanding its clinical indications [13].

## Case presentation

December 2014, a 65-year-old Lebanese male patient known to have severe ischemic cardiomyopathy with left ventricular dysfunction, type II diabetes, hypertension, and chronic moderate renal impairment, was transferred to our cardiac surveillance unit at the Beirut Cardiac Institute (BCI) medical center of Al Rassoul Al Aazam Hospital (RAH) for heart failure management. This tertiary health care center is a community based hospital, located in south Beirut in Lebanon and comprised of 2 community medical centers with a total of 260 beds.

Three months prior to his current admission, the patient had been diagnosed of single vessel coronary disease, which was managed in a peripheral hospital by an angioplasty with drug eluting stent implantation to the left anterior descending artery (LAD). He was then rehospitalized 2 weeks prior to his transfer to our center, in the same peripheral hospital, for acute myocardial infarction and cardiogenic shock. Urgent coronary angiogram showed occlusion of the LAD stent, the other arteries were unremarkable. Thus the patient underwent stent desobstruction, but despite such management his hemodynamics and left ventricular function did not improve, with failure to wean from inotropes. He was then referred to our center.

**Fig. 1:**
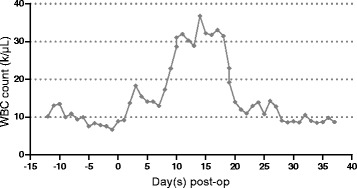
White blood cells count (WBC) following LVAD open-heart surgery in a patient with severe pseumembranous colitis

Upon his admission, the clinical exam revealed hypotension (mean arterial pressure: 65 mmHg), sinus tachycardia (90/min), cold extremities, pulmonary crackles, and hepatomegaly (19.3 cm). Electrocardiogram showed diffuse Q waves and T waves inversion in anterior leads; troponin level was high (1.13 ng/ml; normal range: 0-0.014) and blood test showed elevated creatinine (1.45 mg/dl; normal range: 0.6-1.3). Cardiac ultrasound showed a dilated left ventricle with extensive antero septo apical akinesia and a very low left ventricle ejection fraction (LVEF: 15 %). Chest X-ray showed subacute pulmonary oedema. We maintained the inotropes (Dobutamine), and we inserted an Intra Aortic balloon pump (IABP) on his second day of admission, allowing stabilization of hemodynamics and improvement of diuresis and renal function. Due to the failure of IABP weaning we performed a HeartWare left ventricular assist device (HeartWare, USA) on day 12. Intravenous (IV) vancomycin therapy was started empirically three days pre-operatively (pre-op) and continued post-operatively (post-op) along with imipenem/cilastatin (IV) as infection prophylaxis. The patient was stable initially, with no surgery related complications. On day 4 post-op he developed worsening of his kidney function with creatinine level reaching 2.37 (mg/dl). Vancomycin therapy was stopped. By day 12 post-op creatinine level decreased (1.46 mg/dl). Nevertheless, the patient had high white blood cells count (30.3 k/μL; normal range: 4-11) (Fig. 1); based on empirical evidence *Acinetobacter baumannii* was suspected and he was managed with teicoplanin, anidulafungin and polymyxin E along with imipenem/cilastatin.

By day 14 post-op he developed diarrhea and rectorrhagia (rectal bleeding) and laboratory testing indicated negative results for C. *difficile* antigen. Abdomen and pelvis Computed tomography (CT) scan showed significant thickening of the whole colon and rectum with surrounding fat stranding, illustrating pancolitis which is usually unresponsive to medication and requires frequently surgery [15]. Oral vancomycin and metronidazole (500 mg PO QID) were administered and former medicines were sustained. Day 17 post-op, his WBC decreased but diarrhea persisted, C. *difficile* antigen remained negative; culture stool was negative for *Salmonella* and *Shigella*; *Entamoeba histolytica* cysts (not being the cause behind colitis) were seen in stool and occult blood was positive. He was continued on vancomycin and metronidazole, and started on oral rifaximin, ceftriaxone and probiotics (Euflora). Colonoscopy, performed on day 27 post-op, revealed diffuse colitis, scattered yellowish plaques and large amount of blood throughout the colon. A biopsy from the left colon was taken for histological diagnosis and reported benign colonic mucosa with marked thick layer of acute and chronic inflammatory cells that was most consistent with severe pseumembranous colitis. The patient was thus put on cholestyramine (Quantalan and Questran) regimen. The patient’s condition worsened not responding to any of the conventional therapy. The gastroenterologist prescribed FMT prior to Protocolectomy. On day 32 post-op, FMT (240 cc) was delivered, following the local hospital protocol into the duodenum through gastroscopy; an unrelated healthy person was the donor after proper stool testing for common pathogens. The patient’s diarrhea improved within few hours and clinical resolution occurred after 24 h.

## Conclusions

We describe a case of severe *C. difficile*-associated diarrhea in an advanced heart failure patient who underwent LVAD surgery at our institution and was successfully treated with FMT, a technique used for the eradication of *C. difficile*-diarrhea, which gained much attention recently and proved to be more successful than other conventional treatments. To the best of our knowledge, this is the first local and regional description for FMT as being therapeutically efficient in hospitalized patients. Fecal microbiota transplantation was first described in 1958 for the treatment of pseudomembranous colitis [16], it consists primarily of restoring the normal intestinal flora. Several published cases from USA [17–19], Canada [20], United kingdom [21], China [22–24], Korea [25], Germany [26], Switzerland [27], Hungary [28], Italy [29], Denmark [30], Sweden [31], Norway [32], Romania [33], Finland [34], Australia [9, 35] and Czech Republic [36] have described performing FMT and getting good clinical outcomes. Case reports from France are very rare; in a review article, French authors explain that since there is no standardized procedure for FMT, the matter of considering it strictly in investigational clinical setting must be raised despite its increasing medical interest [37].

Unfortunately, there are minimal studies from the Middle Eastern countries informing about the incidence or the microbiological characteristics of C. *difficile* strains [2]. To date, there have been no reports from the same region describing or recommending the use of FMT as a treatment for severe *C. difficile* infection, instead conventional therapies are employed and surgical procedures are used as a last resort.

Published article, from a Lebanese university hospital, recommended probiotics as prophylactic agents against antibiotics-associated diarrhea [38]. El-Herte et al. from Lebanon reported a case of metronidazole and vancomycin resistant *C. difficile* treated by a combination of rifamixin and tigecycline after the refusal of the patient to undergo surgical procedure [1]. Conversely caution with the use of tigecycline was urged in a Greek case report after its failure to treat a severe *Clostridium* infection in Intensive care unit (ICU) setting [39]. We point out here, that our patient didn’t receive tigecycline due to shortage from medical suppliers during his illness period. A case study from Turkey treated successfully a patient, with end-stage renal disease, having *C. difficile*-associated diarrhea with metronidazole regimen for ten days; justifying their findings about the culture-negative peritonitis by the recent antibiotic therapy, and recommending the consideration of *C. difficile* in patients with culture-negative diarrhea [40]. From Iran, Goudarzi *et al*. have investigated the susceptibility pattern of *C. difficile* clinical isolates and recommended as well metronidazole and vancomycin as first choice drugs for treatment [41]. Also from Iran Sadeghifard et al. recorded susceptibility of *C. difficile* to chloramphenicol and ceftriaxone [42]. Jordanian authors detected the genetic pattern of the *C. difficile*’s toxins among hospitalized patients indicating as well susceptibility towards metronidazole and vancomycin [43]. Similar susceptibility studies were done in Kuwait and Saudi Arabia describing antibiotic resistances without proposing any potential solutions [44, 45]; just one report by Abdulaziz *et al.* informed about the use of intravenous immunoglobulin as adjuvant to antibiotics ensuing with successful outcome [46]. Hospitals in Quatar as well continue on using conventional treatments even for severe cases of *C. difficile* [47, 48]. Published data from Israel reported and recommended the use of conventional antibiotics [49–51] as successful therapy against *C. difficile*. However, despite such guidelines from their country, a recent study from Israel in collaboration with Boston, USA developed frozen FMT capsules for patients with recurrent *C. difficile* infection, and evaluated the safety and effectiveness of such administration [52]. This might open a solution for the standardization of the FMT procedure. We tried to order FMT capsules for our patient, but there were some restrictions from the suppliers’ side, since they require from patients willing to try this treatment to be administered and followed directly by USA providers for investigational purposes.

FMT preparation following the local hospital protocol successfully resolved our patient’s symptoms without recourse to surgery. In conclusion, we recommend fecal microbiota transplantation, for severe forms of *C. difficile* infection, as the best option for treatment.

## Abbreviations

BCI, Beirut Cardiac Institute; CT, computed tomography; ECDIS, European C. *difficile* infection study; ESCMID, European society of clinical microbiology and infectious diseases; FDA, Food and Drug Administration; FMT, fecal microbiota transplantation; IABP, intra Aortic balloon pump; ICU, intensive care unit; IDSA, infectious diseases society of America; LAD, left anterior descending artery; LVEF, left ventricle ejection fraction; PMC, pseudomembranous colitis; RAH, Al Rassoul Al Aazam Hospital.
